# Incongruence of Hemolysis, Elevated Liver Enzyme, Low-Platelet Count Syndrome (HELLP) and Preeclampsia Criteria in Pregnancy: Implications for Medical Education and Obstetrics Training

**DOI:** 10.7759/cureus.67211

**Published:** 2024-08-19

**Authors:** Jacob Jenkins, Aleena A Ferozuddin, Jad Mourad, Zayna Z Abdulla, Angelica Oviedo

**Affiliations:** 1 Physiology & Pathology, Burrell College of Osteopathic Medicine, Las Cruces, USA; 2 Pathology and Laboratory Medicine, Burrell College of Osteopathic Medicine, Melbourne, USA

**Keywords:** constructivism, diagnostic criteria, medical training, obstetrics education, pregnancy-related complications, preeclampsia, hellp syndrome

## Abstract

There is conflicting information in the medical literature regarding hemolysis, elevated liver enzymes, low platelet count syndrome (HELLP) and preeclampsia and whether they are subsets of a single disease or distinct complications of pregnancy. In numerous places, HELLP is described as a severe form or later stage of preeclampsia. However, a detailed medical literature search utilizing NCBI, PubMed, and Elicit: The AI Research Assistant clearly demonstrates that HELLP and preeclampsia are distinct diseases. While they share similarities, each one has unique diagnostic criteria, pathophysiology, and treatment. We believe that these entities should be taught as separate entities to medical students and residents because this will result in better patient care. Medical educational theories, including constructivism, demonstrate that initial learning experiences heavily influence future learning. The joining of HELLP and preeclampsia in medical school teaching materials is detrimental to students' and trainees' long-term understanding of these two serious complications of pregnancy.

## Editorial

Hemolysis, elevated liver enzymes, and low platelet count syndrome (HELLP), a life-threatening complication of pregnancy, can occur anytime throughout pregnancy or even postpartum. Although HELLP has a prevalence of 0.5%-0.9%, it can cause severe maternal and fetal morbidity and mortality. The maternal mortality due to HELLP is reported to be between 0% and 24%, while the fetal mortality is nearly 37% [1].

Preeclampsia is also a serious pregnancy complication that can only be diagnosed after 20 weeks gestation and is characterized by high blood pressure and damage to maternal organs. This condition can also be life-threatening for both mother and fetus if left untreated. Preeclampsia is a common gestational complication with a prevalence of approximately 2%-15% of all pregnancies [2]. The maternal mortality rate is estimated between 1% and 30% worldwide [3].

Given that HELLP and preeclampsia are both serious complications of pregnancy, it is important to examine how they are taught throughout medical school and in obstetrics residency. The medical learning theory of constructivism states that students “construct” new knowledge by assimilating new material into what they already know [4]. Indeed, clinical diagnostic reasoning depends on constructivist processes. When a patient presents with a problem, the physician evaluates the history and physical based on their mental models of illness presentation that they have built up during prior training. Of course, if those mental models are incorrect, the next step of obtaining additional investigations such as blood tests will also be incorrect. The final diagnosis may then be erroneous due to the incorrect mental model of illness [5]. It is critical that students learn clinical content correctly in the first two years of medical school so that they can correctly assimilate new clinical knowledge in clerkship years and residency.

Upon thorough investigation, many of the resources used to educate medical students and obstetrics residents teach HELLP as a complication or subset of preeclampsia. However, we recommend that these entities be clearly distinguished in undergraduate medical education and obstetrics residency. In this editorial, we will explore the differences between HELLP and preeclampsia and recommend that these be taught as separate entities.

We conducted a literature review as follows. NCBI and PubMed were searched using the following words and phrases: ‘HELLP Syndrome,’ ‘Preeclampsia,’ ‘HELLP Outcomes,’ ‘HELLP Incidence and Prevalence,’ ‘Preeclampsia Mortality Rate,’ ‘Preeclampsia versus HELLP,’ ‘HELLP Syndrome precursor,’ ‘HELLP Syndrome in Preeclampsia,’ and ‘HELLP Syndrome and risk factor.' Chapter 18 of the textbook 'Robbins and Cotran Pathologic Basis of Disease 10th Edition' [6], Chapter 40 of the textbook 'Williams Obstetrics 26th Edition' [7], and Chapter 5 of 'Obstetric Intensive Care Manual 5th Edition' [8] were also read for a better understanding of HELLP and preeclampsia. Elicit: The AI Research Assistant was prompted using the following phrases or questions: ‘HELLP syndrome without hypertension’, ‘HELLP syndrome with multipara women’, ‘Can HELLP syndrome occur without hypertension’, ‘HELLP syndrome in multiparous women’. The database UptoDate [9] was searched using the phrase: ‘Preeclampsia and HELLP’. The references that resulted from these searches were reviewed and analyzed for their relevance to this subject.

Medical hypothesis

HELLP is frequently taught as a subset or complication of preeclampsia. Although both are complications of pregnancy, HELLP is a distinct clinical entity rather than a subset of preeclampsia. HELLP has different diagnostic criteria, pathophysiology, and treatment when compared to preeclampsia.

Support for hypothesis

Knowledge Sources Utilized by Medical Students and Obstetrics Residents Regarding HELLP and Preeclampsia

We reviewed multiple resources commonly used by medical students and obstetrics residents regarding HELLP and preeclampsia. For example, the major pathology textbook used in many US medical schools, 'Robbins and Cotran Pathologic Basis of Disease' [6], notes that a consequence of preeclampsia or eclampsia is HELLP. The book states that this may be due to various placental factors released. However, Robbins does not claim that elevated blood pressure is necessary for HELLP but notes that HELLP can be a consequence of preeclampsia, which does require the presence of elevated blood pressure. The professional association, American College of Obstetrics and Gynecology (ACOG), notes that 'Preeclampsia can cause HELLP,' on its website [10]. Additionally, 'Williams Obstetrics 26th Edition' [7] reports that preeclampsia can occur with HELLP, which typically results in worse outcomes. 'Williams Obstetrics 26th Edition,' however, did not elaborate on the possible causes of HELLP compared to preeclampsia. Another obstetrical textbook utilized by obstetrics residents, 'Obstetric Intensive Care Manual' [8], stated that HELLP is a severe form of preeclampsia and that the diagnosis may be hard to elucidate because blood pressure may be slightly elevated. It also noted that a diagnosis of HELLP is automatically a manifestation of severe preeclampsia. The classification of HELLP as a subset or complication of preeclampsia in study resources used in medical training leads to confusion since increased blood pressure is the sine qua non of preeclampsia but not of HELLP. We have summarized these educational resources in Table 1.

**Table 1:** Comparison of HELLP and preeclampsia sections in various educational resources Commonly used educational resources do not describe HELLP and preeclampsia as separate entities.

Educational resource	Section on HELLP	Section on preeclampsia	Does the resource denote them as separate entities?
Robbins and Cotran Pathologic Basis of Disease, 10th Edition	Yes	Yes	No
Williams Obstetrics, 26th Edition	Yes	Yes	No
Obstetric Intensive Care Manual, 5th Edition	Yes	Yes	No
ACOG website	Yes	Yes	No
UpToDate website	Yes	Yes	No

HELLP and Preeclampsia Diagnostic Criteria

According to the Tennessee Classification System, the criteria for diagnosing HELLP include 'haemolysis with increased LDH (>600 U/L), AST ≥ 70 U/L), and platelets < 100 x 109/L.' [11]

Preeclampsia criteria include a systolic blood pressure of ≥140 mmHg or diastolic blood pressure of ≥90 mmHg on two separate readings at least four hours apart, or shorter interval timing of systolic blood pressure of 160 mm Hg or more or diastolic blood pressure of 110 mmHg or more, all of which must be identified after 20 weeks of gestation [12]. The criteria for the diagnosis of HELLP versus preeclampsia are shown in Table 2.

**Table 2:** Diagnostic criteria for Tennessee HELLP Classification and Preeclampsia Summary of the diagnostic criteria for Tennessee HELLP Classification and Preeclampsia shows no overlap in criteria.

Diagnostic Criteria Summary	HELLP	Preeclampsia
LDH (Lactate Dehydrogenase)	LDH≥ 600 IU/L	Not a criterion
AST (Aspartate Aminotransferase)	AST ≥ 70 IU/L	Not a criterion
Platelets	Platelets ≤ 100,000/ μl	Not a criterion
Systolic blood pressure	Not a criterion	≥140 mmHg
Diastolic blood pressure	Not a criterion	≥90 mmHg

The application of constructivist processes to medical student education has been shown to improve outcomes. A hospital-to-home transition of patient care workshop intervention that used constructivist theory improved medical students' knowledge, skills, and attitudes. That study showed that students exposed to the constructivist intervention had improved knowledge of the role of medication errors as a source of post-discharge adverse outcomes [13]. A separate study showed a 38% reduction in prescribing errors in a population of medical interns that underwent a constructivist informed feedback intervention designed to prevent prescribing errors [14].

Clinical Aspects of HELLP and Preeclampsia

Patients with HELLP typically present between 28 and 37 weeks of pregnancy or postpartum, within seven days of delivery [1]. In contrast, preeclampsia begins after 20 weeks of gestation [2].

HELLP is likely a manifestation of systemic inflammation and complement activation. Irregular placental development likely triggers this inflammatory response. The exact mechanism behind this reaction is yet to be fully understood. Additionally, a minority of HELLP cases have been linked to complement dysregulation resulting from preexisting thrombotic microangiopathies, manifesting with symptoms similar to hemolytic uremic syndrome [1]. The abnormal inflammatory factors cause apoptosis or necrosis of the maternal liver sinusoidal endothelial cells, which contributes to the elevated liver enzymes and hemolysis classically seen in HELLP [15].

In contrast, preeclampsia results from abnormal placentation with resulting placental endothelial dysfunction [12]. The clinical findings include new-onset hypertension, which may be accompanied by proteinuria. The underlying pathophysiology likely results from uteroplacental ischemia, which causes the release of a toxin, which then causes maternal hypertension. The disease can progress and result in multi-organ damage of the maternal liver, kidney, and brain [16]. 

Since the management for HELLP and preeclampsia are different, diagnostic criteria must be followed in order to provide effective treatments. The therapy for HELLP is focused on organ dysfunction [9], while preeclampsia treatment is heavily focused on the control of hypertension [8].

Conclusion

The initial presentation of educational content in the early years of medical education heavily influences later learning and consolidation of knowledge. Constructivist educational interventions have been shown to improve learning and outcomes in medical students and interns. As medical students and obstetrics residents progress in their careers, these initial experiences will contribute to the type of care they provide to their future patients. Commonly used resources demonstrate conflicting information regarding HELLP and preeclampsia. In addition, many research papers display inconsistency in distinguishing HELLP and preeclampsia. These diseases have distinct etiologies, pathophysiologies, and prognoses. We propose that these pregnancy complications should be taught as distinct entities since they have differing presentations, diagnostic criteria, and treatments. This has critical implications for the care of pregnant women, ultimately affecting both maternal and fetal outcomes.
